# Differences and Relationships Between Sensory Profile and Repetitive Behavior in Autism

**DOI:** 10.3390/children12040504

**Published:** 2025-04-14

**Authors:** María-Dolores Cárcel-López, Mercedes Ferrando-Prieto

**Affiliations:** 1 Department of Didactics and School Organization, University of Murcia, 30100 Murcia, Spain; 2 AUFREN (Autism Friendly Environment Center), 30107 Murcia, Spain; 3 Department of Developmental and Educational Psychology, University of Murcia, 30100 Murcia, Spain; mferran@um.es

**Keywords:** sensory processing, autism spectrum disorder, repetitive behaviors

## Abstract

Repetitive behaviors are actions that are performed consistently and repeatedly, often without an obvious functional purpose. These types of behaviors are common and diverse in individuals with Autism Spectrum Disorder (ASD) and can influence daily life, making social integration difficult for individuals who experience them and thus becoming a source of distress. Research show that, in most individuals with ASD, sensory processing tends to be affected. It has been suggested that, in children with ASD, sensory processing disorders can cause them to experience their environment in an exaggerated or diminished way, and this affects their behavior. Repetitive behaviors may emerge as a way to regulate the level of stimulation and achieve a state of sensory balance. **Objectives**: The aim of this investigation is to study differences and relationships between the sensory profile and repetitive behavior in autism. **Methods:** A total of 48 students, aged 4 to 26 years (M = 14.39; SD = 7.24), participated. The Bodfish Repetitive Behavior Scale and Sensory Profile 2 by Dunn were used as instruments of measure. **Results:** Results show that the student groups differ only in some variables, with Level 3 students being the most affected. In this sense, Level 3 students present with higher self-injury, greater tactile and movement processing difficulties, and higher registration (passive response and a high threshold of perception). Hyper-reactivity shows a weak prediction power over insistence of sameness, while hypo-reactivity showed none. **Conclusions:** Correlations between repetitive behavior and sensory processing were weak and of low magnitude, which contradicts the previous literature.

## 1. Introduction

Repetitive behaviors (RBs) are actions that are performed consistently and repeatedly, often without an obvious functional purpose. These types of behaviors are common and diverse in individuals with Autism Spectrum Disorder (ASD) [1]. Some examples of these behaviors include body rocking, echolalia (or repetition of words or parts of words); a certain obsession with arranging objects (from largest to smallest, by color, etc.); following strict routines and repeating them frequently; biting their hands or hitting their head; and constantly talking about a topic of interest to them [2,3,4].

These types of behaviors can influence daily life, making social integration difficult for individuals who experience them and thus becoming a source of distress. However, in individuals with ASD, repetitive behaviors can serve a function, as in many cases, they help regulate their emotional states [5,6].

Studies on repetitive behaviors in ASD have distinguished two subtypes of these behaviors: (a) motor and sensory behaviors (RSM), which refer to the repetition of hand or finger movements, and (b) insistence on sameness (IS), which includes restricted interests, rigid routines, and rituals [2,3,4]. The former are known as lower-order behaviors, while the latter correspond to the higher-order domains of repetitive behaviors [7]. 

Some studies have compared the incidence of repetitive behaviors in adults and children, finding that these behaviors decrease with age [3], partly because repetitive behaviors diminish as the mental age of the individual increases [8]. A longitudinal study of high-functioning adults with ASD reported a reduction in repetitive behaviors across five of six domains (stereotyped, self-injurious, compulsive, ritualistic, and sameness behaviors), although restricted behaviors persisted [9]. Similarly, the study by Moore et al. [10] found a significant inverse correlation between age and motor repetitive behaviors in a sample of over 400 adults with ASD. Nevertheless, in that same study, no significant correlation was found between age and insistence on sameness behaviors. The same results were found by Hwang et al. [11]. In addition, other studies have found that in individuals with ASD, both sensory–motor behaviors and insistence on sameness increase with age [12], contrary to what is expected for the neurotypical population. Table 1 offers a summary of the characteristics of repetitive behaviors.

It has been suggested that individuals with autism and a high IQ tend to exhibit behaviors related to sameness, restricted interests, and rigidity in how things are done, whereas individuals with autism and a low IQ are more likely to display lower-level sensory–motor repetitive behaviors [7,8,13]. Bishop et al. [14] suggested that it is the high-level repetitive behaviors (restrictive interests) that are intrinsic or characteristic of autism.

However, the research by Grossi et al. [13] adopting an innovative perspective found that participants with ASD who exhibited simple repetitive patterns did not differ in severity compared to those with ASD who predominantly displayed complex repetitive patterns. The authors defined simple repetitive patterns as those involving a single movement, sensory channel, or vocalization, such as touching an object or producing simple sounds, while complex repetitive patterns were characterized by the combination of multiple movements, sensory channels, or vocalizations, such as sequences of body movements or the repetitive use of words and phrases.

Nwaordu [15] suggested that discrepancies in research may be due to the type of measure used (self-report or observer report, such as from parents), and it was noted that repetitive behaviors may go unnoticed by the observer when they have been adapted to be socially acceptable. This may be the case for some females with ASD, whose interests and stereotyped behaviors are more socially accepted (e.g., they may be obsessed with animals or certain types of products) [16]. In fact, recent research finds no differences in repetitive behaviors in males and females [17].

Some authors have suggested that repetitive behaviors in individuals with ADS serve an adaptive function, acting as a coping mechanism to maintain a homeostatic arousal state, as these behaviors help increase or reduce sensory stimulation [2,6,18]. At this point, it is important to note that for most individuals with Autism Spectrum Disorder (ASD), sensory processing tends to be affected. “Many individuals with Autism Spectrum Disorder (ASD) respond to sensory stimuli in ways that are incongruent with the intensity and nature of sensory stimulation” [19]. Prevalence rates have been reported as high as 86.8% [2]. Some of these manifestations include smelling the rain, discomfort when wearing new clothes that feel like sandpaper, or perceiving the touch of fingers as sharp metal [20]. This atypicality is associated with a deficit in their sensory processing. The deficit in sensory processing has a cascading effect on other areas of development [21,22,23], impacting the adaptation of children with autism.

Sensory integration processing undergoes continuous ontogenetic refinement, facilitating the attribution of meaning to afferent information. The automatization of these processes underlies the infant’s capacity to execute more complex cognitive actions [23]. 

Prior to the integration of sensory information, it must be registered, wherein each stimulus is encoded discretely, and not all systems are activated simultaneously. Following registration, the intensity of perception is evaluated, corresponding to the processes of modulation and regulation. Subsequently, the relevance of the stimulus is distinguished, and its interpretation proceeds. Finally, stimuli deemed significant are integrated, enabling the organization of an adaptive response to contextual demands.

In the integration phase, perception is processed (in the primary sensory cortex, which is the first to receive stimuli from the thalamus: stimulus characteristics), cognition occurs (in the polymodal associative cortex, which is second in the processing hierarchy: assigning meaning to the stimulus), and finally, the emotion in response to the stimulus is processed (in the limbic cortex: rejection or acceptance of the stimulus) to give rise to the adaptive response with all the information.

So, what makes the central nervous system effective in sensory processing is that, within the same neuronal network, the afferent loops of information connect with the efferent loops that activate adaptive responses [24].

**Figure 1:**
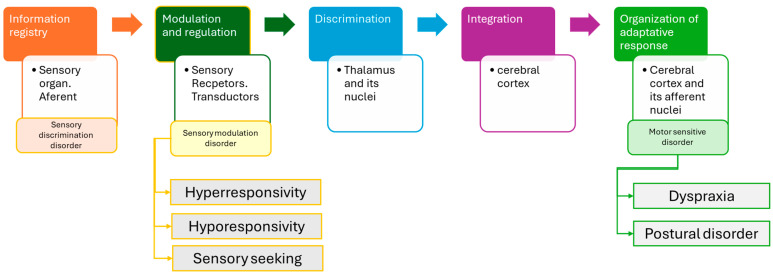
Sensory processing is associated with the neurological structure of each phase and the associated disorder.

The differential idiosyncrasy of each organism leads to variations in the organization and signification of afferent information. Sensory processing dysfunctions (SPDs) affect the various phases of processing (Figure 1), resulting in significant difficulties in achieving optimal adaptive responses.

It has been proposed that children with hypersensitivity are less exposed to physical activity from an early age, and this activity helps develop the foundations of sensory processing [25]. It has been suggested that sensory processing deficits may diminish with age as individuals gain more experience with their surroundings [12,26,27,28], but some longitudinal and cross-sectional studies do not seem to corroborate this hypothesis [10,29].

The relationship between sensory processing characteristics and symptoms of Autism Spectrum Disorder (ASD) shows considerable variability among individuals with this diagnosis [7]. This diversity is a key feature of sensory symptoms in ASD, with reactions ranging from strong sensory overload in response to a stimulus to a complete lack of reaction to the same stimulus in different individuals [30,31,32].

Moreover, this variability is not only interindividual but can also occur within the same individual (intraindividual), depending on the person’s emotional and physical state [33,34]. Some adults with ASD have reported that their sensory reactivity varies depending on their mood and stress levels. For example, when they feel relaxed and well rested, their tolerance to stimuli such as sounds, touch, or bright lights is higher; however, in situations of fatigue or stress, those same stimuli can become more distressing and difficult to tolerate [35].

Repetitive behaviors, as well as difficulties in sensory processing, are key characteristics of Autism Spectrum Disorder (ASD), and some studies have attempted to understand how these two factors are interrelated. Research suggests that, in children with ASD, sensory processing disorders can cause them to experience their environment in an exaggerated or diminished way, and this affects their behavior, as repetitive behaviors may emerge as a way to regulate the level of stimulation and achieve a state of sensory balance [4,18].

The sensory profile of each individual can influence the intensity and type of repetitive behaviors they exhibit. For example, in those with low auditory sensitivity, it is common to observe them seeking additional stimulation through activities such as hitting objects or making repetitive vocalizations [36,37]. This suggests that repetitive behaviors can serve different functions, ranging from increasing stimulation to reducing anxiety, depending on the individual’s sensory profile [4,38]. Furthermore, these behaviors could act as a self-regulation mechanism in response to sensory overload, reducing the individual’s level of arousal [22]. In this context, repetitive behaviors can be seen as a strategy to reduce environmental uncertainty when these modulating references are lacking [25,39]. 

An additional theory proposes that the unique sensory processing characteristics of individuals with ASD may be linked to a reduced influence of prior experiences, making it more difficult to adapt to new situations.

Recent studies confirm a relationship between repetitive behaviors and sensory characteristics, even after adjusting for factors such as age and intellectual quotient [7].

Although there is consensus regarding the connection between sensory alterations and repetitive behaviors in ASD, it has not yet been established whether these are distinct phenomena or manifestations of the same underlying process [22].

Despite the relevance of this topic, only about twenty studies have investigated the relationship between these two constructs. These investigations were conducted with samples of students with ASD of varying severity; some studies used samples with average IQ [11,15,40,41,42], while others focused on students with lower levels of functioning [8,30,43]. It is worth mentioning that early research on this topic began in 2005, before the 2013 APA revision [44] included sensorial processing disorders as an identifying factor and established severity levels for ASD. Nowadays, the standard classification is as follows: Level 1—requires support (approximately equivalent to the previous diagnosis of Asperger’s), Level 2—needs substantial support, and Level 3—requires very substantial support, corresponding to severe ASD in the previous classification.

Most studies have been conducted with children and adolescents, with the exception of the work by Nwaordu et al. [15] and Gonthier et al. [30], who used samples of adults. The most commonly used scales in these studies to measure repetitive behaviors were the Bodfish scale and the RBQ-2 scale.

Sensory processing was measured using scales based on Dunn’s model: earlier studies used scales developed by McIntosh et al. [45,46], while others employed Dunn’s questionnaires (the Adult Sensory Profile, Sensory Profile 2). In addition, some studies used instruments specifically developed for their purposes, such as The Glasgow Sensory Questionnaire (GSQ) [47]; the Sensory Preferences Questionnaire (SPQ) [48]; and the Sensory Questionnaire (SQ) [49].

In nearly all cases, statistically significant correlations were found between repetitive behaviors and sensory processing, typically ranging between 0.4 and 0.7. The nature of these correlations varies from study to study, as does their magnitude, with the highest correlations found between total sensory processing scores and repetitive behaviors (e.g., Black et al. [40] [r around 0.7] and Fetta et al. [22] [rho = 0.8]), with Gal et al. [18]’s study being an exception.

Due to the complexity of both constructs, many studies report only a general correlation [8,41,50]; others report correlations using the main factors (sensory movement and insistence on sameness for repetitive behaviors and hyper- and hypo-reactivity for sensory processing) [4,40]. Studies analyzing sensory systems are exceptional [51]. In many studies, the subscales or dimensions of repetitive behavior are not differentiated. In those where a distinction is made between sensorimotor repetitive behaviors and insistence on sameness, the results regarding which dimension is more affected by the sensory profile are inconclusive. In some cases, higher correlations are found between sensory processing and insistence on sameness [7,40], while in others, higher correlations are found between sensory processing and sensorimotor repetitive behaviors [18,41,52]. These correlations cannot be attributed, at first glance, to the sample selection of the studies. A summary table of this review can be found in Appendix A.

Our aim is to explore the relationship between sensory processing and repetitive behaviors in a Spanish sample, because, to the best of our knowledge, this has not been studied in Spain. In addition, we will use a heterogeneous sample, addressing one of the main flaws in previous studies, which rarely contemplate the full spectrum.

## 2. Materials and Methods

The participants in this study were recruited from two previous studies conducted by Cárcel López. One of those studies aimed to explore the effects of a sensory processing intervention in children with ASD [53,54], while the other aimed to study the relationship between eating and sleeping habits with sensory processing and repetitive behavior [55]. The participants attended either a semi-private school in the Region of Murcia or a residential facility specifically designed for adults with ASD. Ethical approval for this study was granted by the University of Murcia’s ethics committee.

Participants had been diagnosed with ASD by the Child and Adolescent Mental Health Center of the Region of Murcia after being referred from the educational center by the Specific Educational and Psycho-pedagogical Guidance Team for Autism and Other Severe Developmental Disorders of the Department of Education (this is a specific team among the school support services provided by the local authority). This diagnosis was made at an early age for all participants in our sample. Once the Educational Guidance Teams were informed about the diagnosis, the students were enrolled according to their support needs, either in integration (mainstream settings with support) or in Specialized Open Classrooms (self-contained classrooms for those students with high support needs, offering inclusion opportunities). Our sample includes students from both educational settings. In addition, students over 21 years old who could attend a school were recruited from a daycare center specialized in individuals with ASD. Most individuals at this center presented with Level 2 and Level 3 ASD.

A total of 48 students, aged 4 to 26 years (M = 14.39; SD = 7.24), participated. Of these, 8 presented with Level 1 severity, 16 with Level 2, and 24 with Level 3. Table 2 displays the distribution of participants by age and severity level. For some students, the Bodfish and PS-2 scales had been administered on more than one occasion. The first measurement taken was consistently used.

### 2.1. Data Analysis

The data analysis of this study was based on descriptive statistics techniques (means, standard deviations) and group comparison, using the Kruskal–Wallis test due to the sample size and the non-normal distribution of the data. For the same reason, Spearman’s rho correlation index was used instead of Pearson’s. In order to examine the predictive power of sensory processing on repetitive behaviors, quantile regression analyses were performed. The data were analyzed using the statistical software package SPSS v. 28 for Windows.

There were no missing data in this dataset, so no data processing was necessary.

Two instruments were used for this study: the Repetitive Behavior Scale by Bodfish and the Sensory Profile by Dunn.

### 2.2. Escala de Conductas Repetitivas de Bodfish (Bodfish Repetitive Behavior Scale, BRS)

This scale aims to measure and classify repetitive behaviors in individuals with Autism Spectrum Disorder and other developmental disabilities. It is designed to capture both the frequency and intensity of repetitive behaviors across various categories, including the following: (a) repetitive motor behaviors—repetitive body movements, such as rocking, hand-flapping, or tapping; (B) routines and resistance to change—preference for repeating specific activities and difficulty adapting to changes in the environment or daily routines; (C) obsessive interests and preoccupations—excessive interest in certain topics or activities; (D) object manipulation—tendency to arrange, align, or manipulate objects in a fixed and constant manner.

The BRS is based on direct observation and reports from caregivers or family members, who rate the intensity and frequency of these behaviors to obtain a profile of repetitive behavior.

The scale has been adapted to the Spanish context and has demonstrated strong psychometric properties. Specifically, “the Total RBS-R had an α = 0.97, which is equivalent to a very high test–retest correlation; stereotypic (ICC = 0.97), self-injurious (ICC = 0.98), compulsive (ICC = 0.97), ritualistic (ICC = 0.96), sameness (ICC = 0.97), and restricted behavior subscales (ICC = 0.95). In all cases, *p* was less than 0.001” [56].

Given that the literature distinguishes between two main types of repetitive behaviors (sensory–motor vs. insistence on sameness), a principal component analysis with varimax rotation was performed on the subscales, forcing a two-factor solution. According to this analysis, the first component consisted of stereotyped, self-injurious, and compulsive behaviors (corresponding to motor behaviors), and the second component consisted of ritualistic, perseverative, and restricted behaviors (corresponding to sameness behaviors) (see Appendix B). The sensory–motor component accounted for 45% of the variance, and insistence on sameness accounted for 24.5% of the variance.

### 2.3. Sensory Profile 2 by Dunn [57]

The Sensory Profile 2 scale by W. Dunn [57], which was completed by the participants’ teachers, is an 86-item test assessing the participants’ behaviors based on their frequency (always, often, half the time, occasionally, or not applicable). The teachers who completed the scale were well familiar with the students, as they had been together for at least 2 school years.

This test offers three types of scores related to the following:

Sensory systems (auditory, visual, tactile, movement, body position, and oral).

The sensory processing pattern. Dunn [57] proposed a sensory processing model that is based on modulation thresholds (high or low) and the response strategy (passive or active). According to the combinations of response strategies and modulation thresholds, we can find 4 profiles of students:

(1) “Seekers” are those who have an active response and a high threshold;
(2) “Avoiders” are those who have an active response and a low threshold;
(3) “Sensitives” are those who have a passive response and a low threshold;
(4) “Bystanders” (registration) are those who have a passive response and a high threshold of perception.

School factors refer to the following: (1) whether they need external help to participate in learning; (2) whether they show awareness of and attention to their learning environment; (3) their tolerance of the environment; and (4) their willingness to learn.

For each of the scales, the test provides score ranges categorized as “same as others”, “more than others”, “much more than others”, “less than others”, and “much less than others”. The scale has good internal reliability (alpha of the scales ranging between 0.72 and 0.90), as well as adequate test–retest and inter-rater reliability. In addition, the instrument has good validity, as shown by the correlation indices with other tests [57].

In the work of [8], the “Behavior” scale and its items were considered similar to the items of the Bodfish Repetitive Behavior Scale, and therefore, the authors chose to exclude them from the analysis. Taking this into consideration, we adopted the same approach.

This model was chosen because, after reading the previous literature, it appeared to be the most influential one, based on McIntosh et al. [45]’s work.

## 3. Results

### 3.1. Differences in Sensory Processing and Repetitive Behavior Depending on ASD Level

First, descriptive statistics are provided for the Sensory Profile 2 variables and the Repetitive Behavior Scale scores, for the entire sample and for each group of students based on their ASD severity (Table 3). According to the Kruskal–Wallis tests for independent samples, the student groups differ only in some variables: tactile processing, movement processing, registration, and self-injurious behavior. The differences are found only between Levels 3 and 2. Another interesting result is found: the same differences are not observed between Levels 3 and 1. Thus, it could be suggested that IQ or developmental level is not a key factor in the manifestation of repetitive behaviors or sensory processing difficulties.

We considered that our results may be mediated by the age of participants, as they belong to a broad spectrum of ages. Due to our sample size, it would not be appropriate to conduct parametrical analyses such as MANCOVA, which would have allowed us to control for the effect of age while examining differences based on ASD severity level. Even so, and out of curiosity, we performed such an analysis, finding that auditory, visual, and behavior processing was affected by age, as were avoidance and registration. When controlling for the influence of age, differences based on ASD level were found for tactile processing and registration.

### 3.2. Relationship Between Sensory Processing and Repetitive Behavior

Second, we present the correlations between the Sensory Profile variables and repetitive behaviors, analyzed both by sensory system (Table 4) and by quadrant (Table 5). Subsequently, the influence of the items from the “Behavior” scale of Sensory Profile 2 was considered for removal; therefore, the avoidance, sensitivity, and registration dimensions are presented without these items.

As expected, the items on the behavior scale show the highest correlations with repetitive behaviors, although the correlations are of medium–low magnitude. The correlations with auditory and visual (which have traditionally shown high correlations with repetitive behaviors) do not yield high coefficients. Only one statistically significant correlation is found between auditory and perseveration (rho = 0.318), which is of low magnitude.

Regarding the correlations with the Sensory Profile quadrants, we observed that sensory avoidance behaviors are primarily related to sameness behaviors such as perseveration and rituals, as well as self-injurious behaviors. Sensory-seeking behaviors are positively related to stereotypies and sensory–motor repetitive behaviors (see Table 5). A positive and moderate relationship was found between the SP2 avoidance pattern and perseverative and sameness behaviors on the RBS-R. The relationship between perseveration, avoidance, and sensitivity appears stable even when removing the items on the “Behavior” scale.

Finally, a regression analysis was performed to examine whether sensory processing, specifically hyper-reactivity and hypo-reactivity, can predict repetitive behaviors. To achieve this, quantile regression was applied. Two separate regression analyses were conducted: one using sensory movement behaviors as the dependent variable, and the other using insistence on sameness as the dependent variable. The results, presented in Table 6, indicate an increasing predictive power of hyper-reactivity over insistence on sameness. Specifically, the coefficients are significant for the 50th quantile (the mean), explaining 12% of the variance, and for the 75th quantile, accounting for 18% of the variance. Repetitive behaviors associated with sensory-movement are not predicted for hyper-reactivity or for hypo-reactivity, meaning that they may be less affected by sensorial processing.

## 4. Discussion and Conclusions

Our study aimed to explore the connection between two closely linked constructs in ASD: repetitive behaviors and sensory processing deficits. To our knowledge, this type of study has not been conducted before with a Spanish sample.

Those who live and work with individuals with autism are well aware of certain characteristic behaviors, such as covering their ears in response to loud noises, walking in circles, rocking while seated, repeatedly singing the same song, and, in some cases, engaging in self-injurious behaviors. These and other repetitive, persistent actions are often observed in everyday interactions.

On the other hand, while most people with ASD suffer from sensory processing disfunction (90%) [58], not all individuals with autism show repetitive behaviors, either in quantity or quality.

Our results regarding differences in sensory processing across ASD severity levels show minimal statistical differences. The ones found are in tactile processing, movement, and registration. This may indicate that sensory processing difficulties are intrinsic to the ASD condition.

From a theoretical standpoint, the link between these behaviors can be viewed as a cause-and-effect relationship: sensory processing difficulties may lead to maladaptive behaviors, including avoiding overwhelming stimuli or actively seeking sensory input to regulate their environment and improve interaction. Another perspective suggests that anxiety may mediate this relationship—atypical sensory experiences trigger anxiety, which in turn fuels repetitive behaviors. Despite these theoretical explanations, empirical evidence remains limited. While many studies report significant correlations between sensory processing and repetitive behaviors [8,18,22,40,41,42,59], the specific patterns of these correlations vary. For instance, Fetta et al. [22] found a correlation of approximately 0.8, though it has been suggested that this high value could stem from overlapping items between repetitive behavior scales and sensory processing measures. Gabriels et al. [8] attempted to address this issue by removing items related to repetitive behaviors from sensory processing assessments yet still found moderate correlations (r ≈ 0.5), a result similar to that reported by Chen et al. [42] and Bart et al. [59].

When examining the quadrants proposed by Dunn [57], our findings indicate that repetitive behaviors related to insistence on sameness are more strongly associated with hypersensitivity (avoidance and sensitivity quadrants), whereas sensory-movement-related repetitive behaviors are linked to sensory-seeking tendencies. These results are consistent with the findings of Black et al. [40] and Noda et al. [7], who also reported higher correlations between sensory processing and insistence on sameness.

Further statistical analysis indicates that while repetitive behavior may be predicted based on sensory processing, interestingly, only sameness appears to be significantly associated with sensory processing, whereas repetitive sensory movements are not. In the previous literature, predictions were made using the total score of each variable, with the explained variance ranging from 61% [22] to 20% [59]. According to our calculations, hyperresponsivity can account for 12% to 18% of the variance of insistence on sameness. This gives us a clue on how to approach behavioral problems with ASD students: interventions based on sensory processing (e.g., Snoezelen or sensory integration) may improve students’ repetitive behaviors [53].

Based on our results, we could state two things: (1) Sensory processing affects individuals across the ASD spectrum equally. (2) There may be a causal relationship between sensory processing and repetitive behavior.

Interpreting these results leads to the following inference: what is characteristic of ASD individuals is the insistence on sameness, whereas repetitive behavior may be more affected by IQ or mental development. Balancing their bodies, flapping their hands, etc., may be a response to other factors not associated with sensory processing difficulties but with some mechanism to deal with anxiety.

Our results should be taken with caution, as unlike the previous literature, our correlation indices appear unusually low. This may be due to idiosyncratic characteristics of our participants that differentiate them from those in other studies. Upon initial examination, this difference cannot be attributed to sample size, as other studies have used sample sizes similar to ours [4,22,25,42,43,59]; we speculate that this difference could be due to our sample including individuals with greater levels of impairment. In this regard, studies involving low-IQ participants also reported weaker correlations [28]; but still, the correlation pattern in our study does not align with prior research, which has found stronger associations with auditory [7,60], movement-related [7], and tactile processing [60]. We may speculate, although it is a bold idea, that the difference lies in the environment in which the participants live. In our case, both the school and the residential center are clearly oriented to the needs of students with ASD. These are contexts where these individuals carry out their normal activities in a calm, safe environment, where they know their caregivers and the routines have been previously established. Another possibility is a problem with observers: teachers may have difficulty differentiating between different constructs and may tend to give generally higher or lower scores for each participant.

Overall, our findings support the idea that repetitive behaviors in ASD are somehow intertwined with sensory processing. Additionally, they suggest that the nature of this relationship varies depending on the specific type of repetitive behavior and the individual’s sensory profile.

Research in this area presents significant challenges, as different studies adopt distinct approaches to analyzing sensory profiles—some categorize them by sensory modality, while others use broader quadrant-based frameworks. Studies with bigger sample sizes and participants in different contexts can give us a more realistic picture; in addition, longitudinal research, although much more costly and difficult, can provide us with valuable information on the developmental trend of both constructs and their relationship. A meta-analysis could be valuable in synthesizing these varied findings, providing a clearer understanding of the underlying patterns in the data we have.

## Figures and Tables

**Table 1:** Summary of the characteristics of different repetitive behaviors.

	Subtypes	Corresponding Bodfish Scales	Relationship with IQ	Relationship with Age
Repetitive Behaviors	Insistence on Sameness	Stereotyped, self-injurious, and compulsive	Related to high IQ, with higher levels of adaptation	Decreases with age
	Sensory and Motor Behaviors	Ritualistic, sameness, and restricted	Related to low IQ and poorer adaptive levels	Do not appear to be affected by age

**Table 2:** Students’ distribution by severity level and age.

	Level 1	Level 2	Level 3	N
26 y.o.*		1	1	2
24 y.o.			3	3
23 y.o.		1	2	3
22 y.o.		2	2	4
21 y.o.		3		3
20 y.o.			3	3
18 y.o.			1	1
16 y.o.	1		2	3
15 y.o.		1		1
14 y.o.		2	1	3
13 y.o.		1	1	2
12 y.o.	1			1
11 y.o.		1		1
10 y.o.			1	1
9 y.o.	1			1
8 y.o.	1		1	2
7 y.o.	1		2	3
6 y.o.	2	3	1	6
5 y.o.	1	1	1	3
4 y.o.			2	2
**Total**	**8**	**16**	**24**	**48**

* y.o.: years old. Level of ASD severity according to DSM-5; N = number of students by age; Total = total number of students by ASD severity level.

**Table 3:** Differences in sensory profile and repetitive behaviors depending on ASD Level.

	Group	Min–Max	Mean	DT	Median	Mode	Kruskal–Wallis	Post Hoc
Stereotyped B.	G1	0–8	2.38	2.72	2	0	χ^2^(2, 48) = 5.672; *p* = 0.059 ε^2^ = 0.082	
	G2	1–12	3.44	2.99	2	2		
	G3	0–13	5.38	3.65	5.5	4		
	All	0–13	4.23	3.45	3	2		
Self-injurious B.	G1	0–6	1.88	2.30	1	0	χ^2^ (2, 48) = 8.194; *p* = 0.017 ε^2^ =0.138	G2 vs. G3 ↑
	G2	0–6	1	1.75	0	0		
	G3	0–12	3.67	3.41	3	0		
	All	0–12	2.48	3.00	2	0		
Compulsive B.	G1	0–7	2.88	2.59	2	2	χ^2^ (2, 48) = 2.12; *p* = 0.347 ε^2^ =0.003	
	G2	0–10	2.81	2.95	2.5	0		
	G3	0–13	4.04	3.29	4	5		
	All	0–13	3.44	3.07	3	0		
Ritualistic B.	G1	0–11	6.38	4.03	7	6	χ^2^ (2, 48) = 4.426; *p* = 0.109 ε^2^ =0.054	
	G2	0–9	3.69	2.65	4	0b		
	G3	0–10	3.33	3.12	2	0		
	All	0–11	3.96	3.26	4	0		
Perseverative B	G1	0–21	10.25	9.15	9.5	0b	χ^2^ (2, 48) = 0.998; *p* = 0.607 ε^2^ =-0.002	
	G2	0–17	5.94	5.40	4	1b		
	G3	0–25	8.58	7.64	7	0		
	All	0–25	7.98	7.27	6	0		
Restricted B.	G1	0–8	3.38	2.50	3.5	4	χ^2^ (2, 48) = 4.695; *p* = 0.096 ε^2^ =0.060	
	G2	0–4	1.75	1.18	2	2		
	G3	0–10	1.75	2.40	1	0		
	All	0–10	2.02	2.14	2	0		
SP Auditory	G1	9–31	21	7.17	21.5	26	χ^2^ (2, 48) = 4.413; *p* = 0.110 ε^2^ =0.054	
	G2	7–29	15.88	6.88	13.5	12		
	G3	8–30	20.58	7.31	23	26		
	All	7–31	19.08	7.36	20.5	26		
SP Visual	G1	12–29	21.5	6.39	21.5	12b	χ^2^ (2, 48) = 1.410; *p* = 0.494 ε^2^ =-0.013	
	G2	10–30	20.75	6.35	19.5	15b		
	G3	7–35	23.46	8.20	24.5	28		
	All	7–35	22.23	7.31	22.5	18a		
SP Tactile	G1	9–21	14.13	4.22	14.5	10b	χ^2^ (2, 48) = 6.250; *p* = 0.044 ε^2^ = 0.09 4	G1 vs. G3 ↑
	G2	9–28	16.06	5.69	15	9b		
	G3	10–29	19.54	5.56	20	20		
	All	9–29	17.48	5.73	16.5	20		
SP Movement	G1	10–29	21.25	5.90	21.5	10b	χ^2^ (2, 48) = 6.108; *p* = 0.047 ε^2^ = 0.091	G2 vs. G3 ↑
	G2	9–34	17	6.49	16.5	9b		
	G3	12–39	22.63	7.63	24.5	12b		
	All	9–39	20.52	7.33	19.5	12a		
SP Behavior	G1	23–43	34.5	8.64	39	23b	χ^2^ (2, 48) = 4.758; *p* = 0.093 ε^2^ =0.061	
	G2	12–42	25.94	8.18	26.5	23		
	G3	15–55	33.96	13.21	29.5	43		
	All	12–55	31.38	11.53	28	23		
SP Seeking	G1	12–21	18.63	3.07	19.5	21	χ^2^ (2, 48) = 2.796; *p* = 0.247 ε^2^ =0.018	
	G2	11–34	19.81	7.57	18	12		
	G3	13–37	22.42	6.85	21.5	14		
	All	11–37	20.92	6.72	20	21		
SP Avoidance	G1	22–53	35.25	10.42	34	22b	χ^2^ (2, 48) = 4.662; *p* = 0.097 ε^2^ =0.059	
	G2	13–43	27.94	8.96	28	18		
	G3	12–58	37.58	15.00	38.5	23b		
	All	12–58	33.98	13.09	31	31		
SP Sensibility	G1	18–43	28.88	9.60	26	38	χ^2^ (2, 48) = 3.567; *p* = 0.168 ε^2^ =0.035	
	G2	13–41	23.81	8.04	23	15b		
	G3	15–42	28.71	8.64	31.5	36		
	All	13–43	27.1	8.74	27	15a		
SP Registration	G1	20–45	37.5	8.98	41	41b	χ^2^ (2, 48) = 6.970; *p* = 0.031 ε^2^ = 0.11	G2 vs. G3 ↑
	G2	14–45	29.94	8.88	29	23		
	G3	16–64	40.17	14.45	43.5	29b		
	All	14–64	36.31	12.69	38.5	29		

Note. G1: ASD students with Level 1 difficulties; G2: ASD students with Level 1 or 2 difficulties; G3: ASD students with Level 1 or 3 difficulties. To control for Type I error, a Bonferroni correction was applied, adjusting the significance level to *p* = 0.00357; ↑ indicates the group with higher scores.

**Table 4:** Correlations between sensory profile and repetitive behaviors for the total sample.

	Hyper-Reactivity	Hypo-Reactivity	PS Auditory	PS Visual	PS Tactile	PS Movement	PS Behavior
Stereotyped	0.127 (0.391)	0.083 (0.575)	0.045 (0.764)	0.034 (0.816)	0.219 (0.135)	0.203 (0.167)	0.090 (0.545)
Self-injurious	0.317 * **(0.028)**	0.188 (0.200)	0.159 (0.281)	0.058 (0.698)	0.233 (0.110)	0.400 ** **(0.005)**	0.245 (0.094)
Compulsive	0.096 (0.518)	−0.018 (0.903)	−0.038 (0.796)	−0.047 (0.753)	0.200 (0.172)	0.027 (0.855)	0.040 (0.788)
Ritualistic	0.303 * **(0.037)**	0.083 (0.577)	0.149 (0.313)	0.011 (0.940)	−0.032 (0.831)	−0.062 (0.676)	0.310 * **(0.032)**
Perseverative	0.494 ** **(<0.0001)**	0.283 (0.052)	0.318 * (0.028)	0.214 (0.144)	0.195 (0.185)	0.136 (0.356)	0.515 ** **(<0.0001)**
Restricted	−0.159 (0.279)	−0.113 (0.444)	−0.151 (0.305)	−0.065 (0.660)	−0.180 (0.221)	−0.128 (0.384)	−0.030 (0.840)
SENSORY-MOVEMENT	0.251 (0.086)	0.134 (0.363)	0.090 (0.543)	0.045 (0.759)	0.289 * **(0.046)**	0.290 * **(0.046)**	0.198 (0.177)
SAMENESS	0.407 ** **(0.004)**	0.200 (0.173)	0.240 (0.100)	0.137 (0.353)	0.098 (0.507)	0.045 (0.760)	0.440 ** **(0.002)**

N = 48; no missing data; *p* values are in brackets; * *p* < 0.05; ** *p* < 0.001; to control for Type I error, a Bonferroni correction was applied, adjusting the significance level to *p* = 0.00102. Significant correlations for *p* = 0.005 have been marked with bold case, and significant correlations at *p* = 0.00102 have been underlined.

**Table 5:** Spearman’s rho correlations between Sensory Profile 2 and repetitive behaviors.

	SP Seeking	SP Avoiding	SP Sensitivity	SP Registration	SP Registration nb ^a^	SP Sensitivity nb	SP Avoiding nb
Stereotyped	0.326 * **(0.024)**	0.127 (0.391)	0.073 (0.623)	0.047 (0.749)	0.085 (0.564)	−0.005 (0.975)	0.179 (0.223)
Self-injurious	0.270 (0.063)	0.317 * **(0.028)**	0.174 (0.238)	0.173 (0.238)	0.226 (0.122)	0.154 (0.319)	0.383 ** **(0.007)**
Compulsive	0.164 (0.264)	0.096 (0.518)	0.056 (0.704)	−0.050 (0.736)	−0.019 (0.897)	0.013 (0.935)	0.092 (0.533)
Ritualistic	0.018 (0.906)	0.303 * **(0.037)**	0.240 (0.100)	−0.051 (0.732)	−0.104 (0.480)	0.173 (0.262)	0.129 (0.381)
Perseverative	0.284 (0.051)	0.494 ** **(0.000)**	0.392 ** **(0.006)**	0.162 (0.270)	0.135 (0.360)	0.311 * **(0.040)**	0.290 * **(0.046)**
Restricted	−0.012 (0.936)	−0.159 (0.279)	−0.093 (0.532)	−0.118 (0.424)	−0.195 (0.184)	−0.127 (0.411)	−0.219 (0.135)
SENSORY-MOVEMENT	0.343 * **(0.017)**	0.251 (0.086)	0.160 (0.279)	0.091 (0.538)	0.146 (0.321)	0.072 (0.644)	0.283 (0.052)
SAMENESS	0.203 (0.166)	0.407 ** **(0.004)**	0.349 * **(0.015)**	0.058 (0.694)	0.019 (0.900)	0.257 (0.092)	0.204 (0.165)

N = 48, no missing values; *p* values are in brackets. * *p* < 0.05; ** *p* < 0.001. To control for Type I error, a Bonferroni correction was applied, adjusting the significance level to *p* = 0.00089. ^a^ nb: dimension without items from the Behavior scale, according to Gabriels et al. [8]’s suggestion that scales may overlap because they share item content. Significant correlations for *p* = 0.005 have been marked with bold case, and significant correlations at *p* = 0.00089 have been underlined.

**Table 6:** Quantile regression coefficients.

	Quantile (τ)	Intercept β (p)	Hyper-Reactivity β (p)	Hypo-Reactivity β (p)	Pseudo R^2^
Dependent variable: SENSORY-MOVEMENT	0.25	0.728 (0.877)	0.194 (0.260)	−0.038 (0.743)	0.028
	0.50	3.185 (0.563)	0.196 (3.28)	−0.023 (0.868)	0.070
	0.75	12.748 (0.151)	0.217 (0.497)	−0.083 (−0.517)	0.056
Dependent variable: INSISTENCE ON SAMENESS	0.25	2.127 (0.709)	0.164 (0.430)	−0.042 (0.764)	0.039
	0.50	5.440 (0.465)	0.544 (0.048)	−0.159 (0.390)	0.121
	0.75	6.231 (0.438)	0.818 (0.0236)	−0.206 (0.300)	0.181

## Data Availability

Data may be available upon request to the authors.

## Abbreviations

The following abbreviations are used in this manuscript:

SP	Sensory profile
ASD	Autistic Spectrum Disorder

## Appendix A

Factor analysis on the dimensions of the Repetitive Behavior Scale.

**Table A1:** Principal Component Analysis of the Dimensions of Repetitive Behaviors.

	I (Sensory–Motor)	II (Insistence on Sameness)
Stereotyped B.	−11	879
Self-injurious B.	77	797
Compulsive B.	376	657
Ritualistic B.	914	92
Perseverative B.	833	191
Restricted B.	782	52
*Explained Variance*	45.083	24.533
*Eigenvalue*	2.705	1.472

## Appendix B

Below is the literature review on the previous research on the relationship between repetitive behaviors and sensory processing in ASD.

**Author**	**Sample**	**Measure PS**	**Measure Repetitive Behaviors**	**Other Measures**	**Results**
Gabriels et al., 2005 [8]	n = 14 ASD. n = 8 high IQ, n = 6 low IQ Age ≈ 10 years	Takes the hyper-reactivity of the Aberrant Behavior Check List	RBS-R; Bodfish et al. [61]	Adaptive behaviors (VINELAND)	*Comparisons*: Differences in RBS based on IQ; by subscale, only differences in similarity. *Correlations*: Negative between RBS and adaptative behaviors; negative between RBS and sleep problems. Significant correlation between RBS and hyperactivity when IQ is controlled. *Predictions*: X
Gabriels et al., 2008 [50]	N = 70 ASD, average IQ 81.4; age from 3 to 19 years	SP, Dunn, [62]	RBS-R; Bodfish et al. [61]		*Comparisons*: Students with more vs. less repetitive behaviors. Did not differ in IQ or puberty level, etc. They did differ in their PS. Those with more repetitive behaviors had more misadjusted PS. *Correlations*: RBS-R and PS significantly correlated, even when excluding the items of the Behavior scale (r ≈ 0.5) and controlling for IQ. *Predictions*: X
Boyd et al., 2009 [41]	n = 61 ASD n = 64 TD Age: 6 to 17 years; IQ > 70	SQ (Boyd & Baranek, [49])	RBS-R; Bodfish et al. [61]	The Behavior Rating Inventory of Executive Function (BRIEF; [63]	*Comparisons*: X *Correlations*: PS (SQ composite score) correlated with stereotypies and compulsions and the total score of the RBS-R. Executive functions: only behavioral regulation correlated with self-injury, compulsions, rituals, and the total of the RBS-R. No significant correlations were found between BRIEF and PS. *Predictions*: X
Chen et al., 2009 [42]	N = 29. Age: 8 to 16 years; IQ > 70	SSP, Dunn [62]	The Childhood Routines Inventory (CRI) [64]	(WISC) The Embedded Figures Test (EFT)	*Comparisons*: Those with difficulties in PS showed greater repetitive behaviors. *Correlations*: Significant correlations between PS and repetitive behaviors (r ≈ 0.5). No significant correlations between EFT and PS. *Predictions*: X
Gal et al., 2010 [18]	Large sample including students with TD and ASD, as well as those with visual loss, auditory loss, and ID	The Short Sensory Profile (SSP; McIntosh et al. [46])	The Stereotyped and Self-Injurious Movement Interview (SSIMI; [65])		*Comparison*s: Students with deficits in PS differ in the prevalence of self-injury. *Correlations*: Strong correlation between PS and self-injury (for the entire sample). *Predictions:* X
Boyd et al., 2010 [66]	N = 67 ASD and n = 42 with developmental delay	SEQ; Baranek, 1999 [67]//SP; Dunn, [62]	RBS-R; Bodfish et al. [61]	The primary cognitive measure was the Visual Reception (VR) Scale of the Mullen Scales of Early Learning [68].	*Comparisons:* X *Correlations:* In general, higher hyperresponsive behaviors were correlated with higher levels of repetitive behaviors in the AD and DD groups. Specifically, significant correlations were found between hyperresponsiveness and the presence of stereotypies, compulsions, and rituals/sameness behaviors. For sensory seeking, a significant correlation was only found for ritualistic/sameness behaviors. *Predictions*: X
Lidstone et al., 2014 [4]	N = 49 ASD, Age: 3 to 17 years	SP; Dunn, [62]	Repetitive Behavior Questionnaire-2 (RBQ-2) [4]. Two dimensions: repetitive motor behavior (RMB) and insistence on sameness (IS)	Spence Children’s Anxiety Scale-Parent Version (SCAS-P; [69])	
Swami, P. R., & Vaidya, P. M. (2015) [52]	N = 30 ASD and n = 30 children with mental deficiency	SPSS Dunn [70]	Behavior problem inventory		In the ASD group, the PS quadrants correlated significantly with self-injury and stereotyped movements. Sensory scores did not show significant correlations with self-injury.
Gonthier et al., 2016 [30]	n = 148 low-functioning ASD n = 148 TD Age 19 to 62 years	AASP; Dunn	E’ chelle Pour l’Observation des Comportements d’Adultes avec Autisme (EPOCAA) [71]	Behavioral Dysexecutive Syndrome Inventory (BDSI)	*Comparisons*: X *Correlations*: Significant correlations around .2 between PS and repetitive *behaviors* (depending on the scale). Predictions: X
Bart et al., 2017 [59]	N = 48 boys, n = 28 with mild disability. Age: 5 to 9 years. (Those with ASD and severe disability were excluded.)	SP, Dunn //SSP, Dunn [62,70]	Childhood Routines Inventory (CRI)//The CRI [64])	Screen for Child Anxiety-Related Emotional Disorders (SCARED)	*Comparisons*: Children with atypical sensory responsiveness had a higher frequency of ritual behaviors and higher levels of anxiety compared with children with typical sensory responsiveness. *Correlations:* Significant correlations around .5 between the Dunn quadrants and repetitive behaviors. *Prediction*s: Anxiety predicts 40% of repetitive behaviors; the sensory profile contributes 20% to the prediction of repetitive behaviors.
Black et al., 2017 [40]	n = 50 ASD; n = 50 TD. Age: 7 to 18 years. Average normal or high IQ	The Short Sensory Profile (SSP; McIntosh et al., 1999) [46]	Repetitive Behavior Questionnaire 2 (RBQ-2; [58]	Spence Children’s Anxiety Scale: Parent Report (SCASP; [69])	*Comparisons: X* *Correlations*: Hypersensitivity correlates with sameness in the ASD group (r = 0.71, *p* < 0.0001), but not in the typically developing group (r = 0.19, *p* = 0.24). *Predictions: X*
Schulz et al., 2018 [51]	N = 114; n = 49 with ASD. Age from 6 to 20 years	SP2; Dunn [72]	(Honey et al., 2012) [73]		*Comparisons*: X *Correlations*: Significant correlations were found between (a) the relationship between repetitive behaviors and sensitivity in ASD and TD groups, and (b) the relationship between repetitive behaviors and sensory sensitivities. Also, significant correlations were found between sensory and repetitive behaviors. *Predictions: X*
Glod et al., 2019 [74]	The parents of 19 children with ASD and 16 children with WS, between 4 and 9 years of age	SP; Dunn, 1999 [62]	Repetitive Behavior Questionnaire (RBQ; Turner, 1995) [65]	Spence Children’s Anxiety Scale—Parent Version (SCAS-P; [69]); Preschool Anxiety Scale (PAS, [75]); Social Responsiveness Scale—Second Edition [76]); Anxiety Scale for Children—ASD, parent version (ASCASD) [77]	*Comparisons: X* *Correlations*: Correlations between PS and repetitive behaviors around .7 (with hypo- and hypersensitivity). *Predictions*: X
Hwang et al., 2020 [11]	N = 292; n = 176 ASD (high functioning?); n = 116 non-ASD	GSQ; Robertson & Simmons, 2012 [47]	Repetitive Behaviors Questionnaire-2 for adults (RBQ-2A; [3])	Intolerance of uncertainty The American Psychiatric Association’s (2013) Severity Measure for Generalized Anxiety Disorder (GAD)	*Correlations*: Positive correlations around .6 between sensory processing and repetitive behavior.
Fetta et al., 2021 [22]	N = 21 + 29 ASD. Age: 3 to 15 years	SSP, Dunn [70]	RBS-R; Bodfish et al., 1999 [61]		*Comparisons: X* *Correlations:* The correlation between PS and total RBS was highly significant (rho = 0.80 (*p* < 0.001). *Predictions:* Non-parametric regression shows that 61% of the variability in total RBS was explained by total PS.
